# We Don't Choose Whom We Love: Predictors for Romantic Attraction to Villains

**DOI:** 10.3389/fpsyt.2022.802988

**Published:** 2022-05-17

**Authors:** Iris Frowijn, Lisa M. W. Vos, Erik Masthoff, Stefan Bogaerts

**Affiliations:** ^1^Department of Developmental Psychology, Tilburg University, Tilburg, Netherlands; ^2^Department of Medical and Clinical Psychology, Tilburg University, Tilburg, Netherlands; ^3^Fivoor Science and Treatment Innovation (FARID), Rotterdam, Netherlands

**Keywords:** intimate partner violence, romantic attraction, maladaptive personality traits, adult attachment style, acceptance of couple violence, women

## Abstract

**Introduction:**

Why are women (not) romantically attracted to dark personalities or villains, which might be a risk factor for intimate partner violence (IPV) victimization? In the current study, it is opted to investigate how adult attachment, maladaptive personality traits, and acceptance of couple violence in women predict romantic attraction to heroic/villainous characters using structural equation modeling (SEM).

**Method:**

First, a pilot study was conducted in 122 heterosexual women (aged 16–25) to select male TV characters. This resulted in the selection of six villains and 10 heroes for the main study, in which 194 other heterosexual women (aged 16–25) were asked to rate the pictures of TV characters through an online questionnaire. This was combined with self-report measures of maladaptive personality traits, acceptance of couple violence, and adult attachment. These variables were entered into a SEM model to assess model fit.

**Results:**

Overall, women rated heroes higher on physical appearance (pilot study) and romantic attraction (main study) compared to villains. We found different direct effects of avoidant (negative) and anxious (positive) attachment styles on romantic attraction to heroes. Moreover, maladaptive personality traits fully mediated the positive effect of avoidant attachment style on romantic attraction to villains.

**Discussion:**

Despite the limitations of the study design (e.g., low *N*, low notoriety of the TV characters), this study emphasizes that women are generally more romantically attracted to heroes (vs. villains). Besides, there are different predictors of romantic attraction to heroes and villains, which requires further investigation, especially in the context of IPV.

## Introduction

In the Netherlands, approximately 5.5% of the adult population has been a victim of domestic violence between 2012 and 2017, which equals ~747,000 people (1). About half of this group is a victim of *intimate partner violence* (IPV), which can be defined as any physically/sexually violent, controlling, or emotionally abusive behavior within an intimate relationship (2). Women are more often victims of IPV than men (1, 2). Previous research has shown that only in about 20% of the incidents, the victimization is reported to the police (3), meaning that the total number of IPV is actually much higher indicating a large dark number (1, 4). The consequences of IPV are extensive and can include multiple mental and physical health problems for both victims and bystanders, such as children witnessing IPV (5, 6).

### Early Predictors of IPV Involvement

A meta-analysis of 124 studies showed an association between childhood experiencing violence/witnessing IPV and IPV involvement (as a victim or perpetrator) in adulthood (7). This phenomenon is called intergenerational transmission of violence, which can be explained by, e.g., the social learning theory (8). It was found that men with violent childhood trauma in particular are more likely to commit IPV in adulthood than women, while women are more often victims of IPV (7). However, this often presumed linear association between childhood trauma and IPV in adulthood is complex (7). Individuals exposed to trauma in childhood are more likely to be hypervigilant to potential danger and develop disruptive cognitive schemas and beliefs about the self and others (9). During the traumatic experiences, victims often engage in “identification with the aggressor” (10), adopting the perpetrator's desires and needs for protective purposes, which can be internalized over time (11). At the same time, these disruptive cognitive schemas can influence the interpretation of others' social (ambiguous) intentions as more hostile, which is referred to as *Hostile Attribution Bias* [HAB; (12)]. HAB can be understood as a general cognitive mechanism underlying aggression, in particular reactive aggression (13, 14). HAB can be activated as a result of environmental cues that trigger emotions and cognitive beliefs (e.g., violence acceptance) (15). Consequently, positive attitudes to violence are an important risk factor for IPV (16, 17), which has been related to a higher rate of actual IPV (both victimization and perpetration) among for instance university students worldwide (18, 19). It is suggested that although the interrelations are complicated and bidirectional, both parent-specific and social risk factors can increase the likelihood of IPV involvement. Consequently this may lead to negative parenting style, child maltreatment and negative outcomes for the child (16). In turn, these negative outcomes include the development of potential risk factors for adult IPV involvement within the child, which is the proposed pathway of intergenerational transmission of violence (16).

Negative experiences in childhood are stored in cognitive schemas that the child develops, which are closely related to the cognitive, affective, and behavioral internal working models that characterize the attachment theory (9, 20). Attachment styles are formed in early childhood by the affective bond with primary caregivers. Through interactions, these attachment-related primary caregivers are internalized in working models of the self and others. This forms the basis for personality development, including the development of both adaptive and maladaptive personality traits (20). Childhood attachment styles are believed to be rather stable affecting further adult relationships throughout life (21). A prominent predictor in the development of insecure attachment styles is the experience of childhood trauma events (22). Childhood trauma and insecure parent-child attachment are positively associated, which may increase the likelihood of developing an insecure adult attachment style. This, in turn, may increase the risk of IPV in adulthood (23). Adult insecure attachment can be divided into two subdimensions, namely anxiety (i.e., fear of rejection and abandonment) and avoidance (i.e., fear of intimacy and closeness) (24). With regard to close relationships, the dimension anxiety represents the internal working model of self (perceived love and acceptability toward self), and avoidance represents the internal working model of other (perceived responsiveness of close relationships) (24). As described in a systematic review, both anxious and avoidant attachment styles have been identified as risk factors for IPV victimization, but mainly with a focus on explaining why individuals stay in an abusive relationship rather than why they feel romantically attracted to someone (25).

### Predictors for Romantic Attraction

Maladaptive personality traits, in particular the Dark Tetrad (narcissism, Machiavellianism, psychopathy, and sadism), have been found to mediate the association between childhood trauma and IPV perpetration in adulthood in both men and women (26, 27). Having a partner with high maladaptive personality traits can increase the risk of becoming a victim of IPV. A systematic review [*N* = 31 studies (28)] even found that certain characteristics (e.g., borderline personality traits) may be present in women that predispose them to become victims of a violent partner relationship. Other research found that schizoid, avoidant, self-destructive, schizotypal, borderline, and paranoid personality traits appear to be more common in women who experienced IPV compared to those who had not (29). This accounts for low self-esteem, emotional dependence, inferiority, and self-blame as well (29). These findings suggest that on the one hand there are risk factors to become a victim of IPV within the characteristics in women, and on the other hand within potential partners through maladaptive personality traits.

This also raises the question: ‘What dynamics can take place within an interpersonal relationship between two intimate individuals and who are (not) attracted to these dark individuals?' In contrast to the multitude of research on personality predictors for romantic attraction, much less attention has been paid to maladaptive personality traits (30, 31). There may be a different effect for men and women, as a speed-date paradigm indicated that women scoring high on psychopathic traits were perceived as selfish, while men scoring high on psychopathic traits were found attractive (32). Likewise, in other studies, similar patterns for women who preferred a man with high levels of maladaptive (psychopathic) personality traits were found (33, 34). Moreover, younger women in particular were found to be more attracted to dark personality traits than older women (35), which might be problematic given the peak of IPV in young adulthood (36, 37). Nevertheless, these correlational findings do not provide insight into why women may be attracted to men with dark personalities. This may be related to relationship goals, as was found that girls (aged 13–16 years) preferred violent boys for short-term relationships and especially non-violent boys for long-term relationships (38). This is consistent with the popular belief that “bad boys” are attractive to some people (e.g., vulnerable individuals), as a positive attitude toward violence is presented socially as more appealing in certain age groups (38). Another explanation could be that psychopathic traits—such as narcissism and fearless dominance—are not always perceived as “bad,” because some psychopathy features show a positive association with heroism and prosocial behavior (39). In addition, it is recognized that in general, similarities attract, especially for similarities on positive personality features (40, 41). However, would this also be the case for people scoring high on maladaptive personality traits? It was found that although participants scoring high on maladaptive traits found dark traits more desirable than participants scoring low on maladaptive traits, they did not find these dark traits particularly desirable. It was suggested that they might be more tolerable but not actively seeking out potential partners with dark personality traits. However, romantic attraction was mainly investigated by a description of traits, which is ecologically not valid for the real world where personality traits are not directly apparent in potential partners (30).

From the perspective of attachment and romantic attraction, everyone would prefer a partner with a secure attachment style to achieve safety (40, 42). This strongly applies to securely attached adults (low scores on anxious and avoidant attachment), who were more likely to prefer partners with a secure attachment style, which can be explained by the similarity principle [i.e., preference for partners with similar attachment styles (40)]. Specifically for women, a secure attachment style was found to be more important than physical attractiveness (whereas this was observed the other way around in males) (42). However, not everyone chooses a secure partner in real life, and these preferences depended on the attachment style of the participant, again observing the similarity principle even for the more insecure attachment styles. Concretely, this means that participants with higher scores on anxiety relatively preferred anxiously attached partners, and women with high avoidance scores relatively disliked partners with low scores on avoidant attachment (42). Therefore, applying this to the context of attraction to dark personalities, it would make sense that women with more insecure attachment styles (high scores on anxious and/or avoidant attachment) would remain in problematic relationships later in life, which would be understood as a continuation of the intergenerational transmission of violence. Therefore, these women would be more romantically attracted to a villain/bad person and less to a hero/good person.

However, other studies have shown that people are generally not attracted to people with maladaptive personality traits (31, 43). Likewise, despite the greater attraction to dark personality traits in young women compared to older women, they preferred men with less dark personality traits (35). An explanation for these inconsistent findings could be methodological. People may be very careful to admit that they are attracted to someone with a dark personality or a villain, as a result of perceived self-threat (44). They suggested that when fictional characters are used in research, it may reduce perceived self-threat and may make people less cautious about favoring those with dark personalities (44). Therefore, we propose to measure romantic attraction by using characters of popular TV series/films, either villains or heroes. However, physical attractiveness is a prominent predictor of romantic attraction (45), not all personality traits are immediately apparent (30), and predicting initial romantic desire—even in a speed-date paradigm—appears to be very complicated (46). Therefore, we propose to use pictures, with the great advantage over a description of traits, that pictures can induce arousal (47).

### Current Study

**Figure 1:**
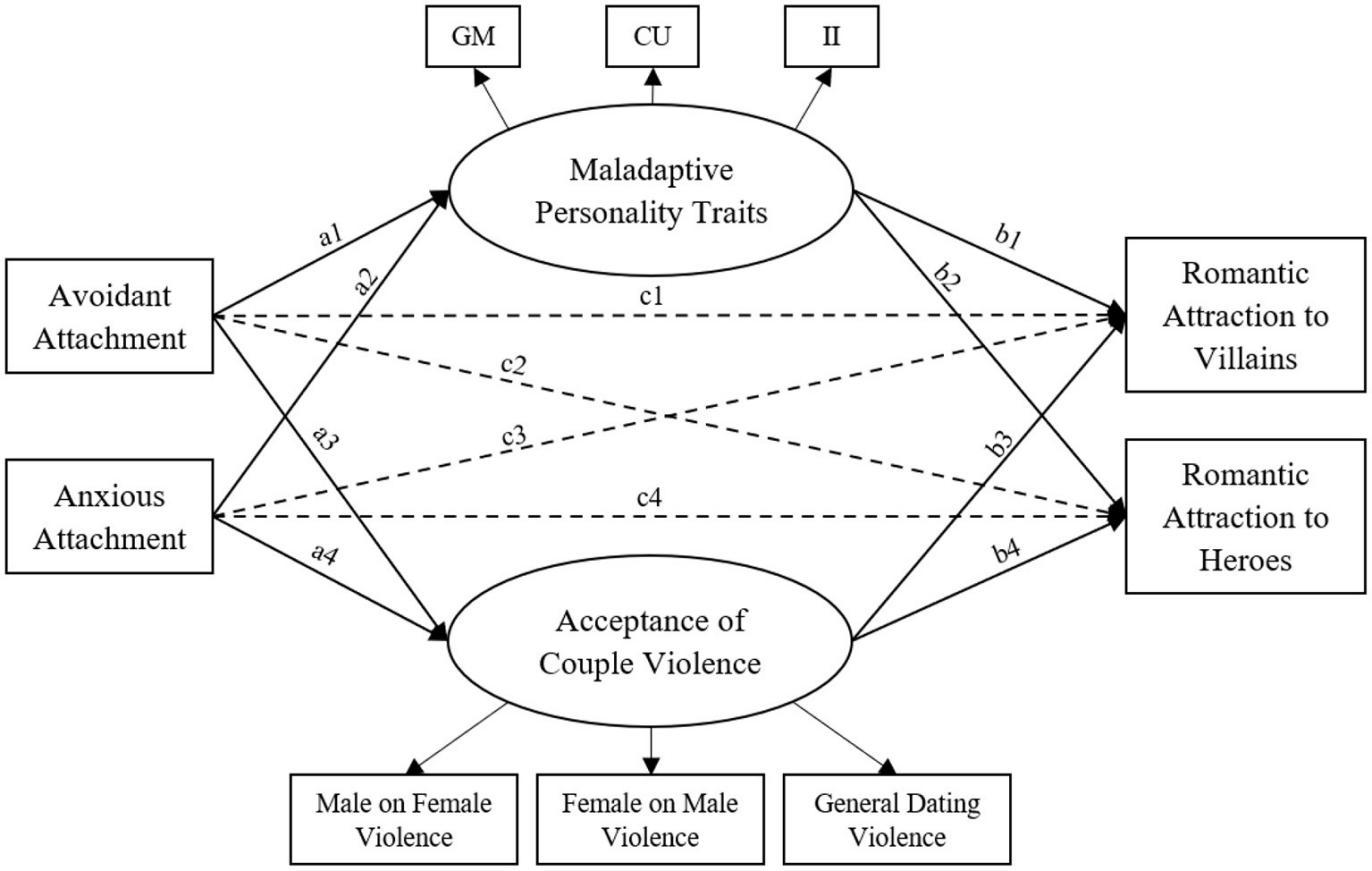
Hypothesized theoretical model predicting romantic attraction to villains and heroes. GM = Grandiose-Manipulative; CU = Callous-Unemotional; II = Impulsive-Irresponsible. Dashed lines represent the direct effects that are expected to be explained by the mediators.

It is important to investigate if and why women are attracted to dark personalities and therefore potentially are more likely to become victims of IPV. The aim of this study was to investigate the effect of adult attachment, maladaptive personality traits, and acceptance of couple violence on romantic attraction to villains and heroes. Instead of using written descriptions of traits, pictures of TV characters were used. To guarantee a reliable selection of these pictures, we first conducted a pilot study. Based on the results we used the selected pictures in the second study to test the theoretical model predicting romantic attraction to villains and heroes (see Figure 1), with the following hypotheses:

H1: Attachment styles are expected to be directly related to romantic attraction (paths c1, c2, c3, and c4). More specifically, higher scores on avoidant and anxiety dimensions are expected to indicate greater romantic attraction to villains (paths c1 and c3). Lower scores on avoidant and anxiety dimensions are expected to indicate greater romantic attraction to heroes (paths c2 and c4) (40).
H2: Maladaptive personality traits are expected to be directly related to romantic attraction (paths b1 and b2). Given that similarities attract, even for maladaptive personality traits (30), we expect a positive effect of maladaptive personality traits and romantic attraction to villains (path b1), which is expected to be negative for romantic attraction to heroes (path b2).
H3: Acceptance of couple violence is expected to be directly related to romantic attraction (paths b3 and b4). Because of the appealing effect of violent boys as opposed to non-violent boys (38), we hypothesize that couple violence acceptance is positively related to romantic attraction to villains (path b3), and negatively to romantic attraction to heroes (path b4).
H4: Adult attachment styles are expected to be directly positively related to maladaptive personality traits (paths a1 and a2), as parent-child attachment forms the basis for the development of personality (20).
H5: Adult attachment styles are expected to be directly positively related to couple violence acceptance (paths a3 and a4). Within an attachment style, an internal working model is formed, including cognitive schemas that take into account attitudes toward violence (9, 20).
H6: We expect that maladaptive personality traits mediate the association between attachment styles on romantic attraction to villains (paths a1^*^b1 and a2^*^b1) and attachment styles on romantic attraction to heroes (paths a1^*^b2 and a2^*^b2).
H7: We expect that couple violence acceptance mediates the association between attachment style on romantic attraction to villains (paths a3^*^b3 and a4^*^b3) and attachment styles on romantic attraction to heroes (paths a3^*^b4 and a4^*^b4).

## Study 1: Pilot Study

### Method

The goal in this pilot was to select reliable pictures of heroes and villains. Participants were gathered through convenience sampling (*N* = 122) and approached online using a Qualtrics questionnaire. Both for the pilot and main study, ethical permission has been granted by the Ethics Review Board of Tilburg School of Social and Behavioral Sciences (RP488), and the data was managed following the General Data Protection Regulation (GDPR) guidelines (http://data.europa.eu/eli/reg/2016/679/2016-05-04). Informed consent was obtained prior to participation, with participants being informed that they would rate TV characters on physical appearance and that the data would be publicly available (anonymously) after the data collection period. Additionally, they were notified that participation would be completely anonymous and voluntary, and that they could withdraw at any time without negative consequences. Inclusion criteria were women with heterosexual romantic preference, aged between 16 and 25 years, and Dutch-speaking. The mean age was 21.60 (*SD* = 2.47; range 16–25), which was significantly higher for women in a relationship (*n* = 71, *M*_*age*_ = 22.32, *SD* = 2.15) than for single women (*n* = 51, *M*_*age*_ = 20.59, *SD* = 2.55), *t*_(120)_ = −4.07, *p* < 0.001. There were no significant differences between women in a relationship vs. single women on level of education (see Table 1, *p* = 0.264). Participants were asked to evaluate pictures on whether they know the TV characters (*yes* or *no*), the physical appearance of the characters on a scale from 0 (*ugly*) to 10 (*beautiful*), and (only if they knew the character) their opinion of whether the characters are good/bad on a scale from 0 (*villain*) to 10 (*hero*). The pictures were preselected by two independent researchers based on the expected notoriety of the TV characters, for which mostly popular recent TV series and films were taken into consideration. It was chosen to select a diverse range of characters regarding ethnicity and physical appearance (e.g., hair color). Because the age range of the participants was set at 16–25 years, relatively young characters were selected. Characters were excluded if they could not straightforwardly be considered good or bad. In total, participants were shown 31 pictures of (preselected) villains and 28 pictures of (preselected) heroes. Moreover, to further control the effects of physical appearance, it was aimed to include the same actor both as villain and as hero (in different TV series/films).

**Table 1:** Education level in Study 1 (Pilot Study).

	**Full sample** ***(N*** **=** **122)**		**Single** **(*****n*** **=** **51)**		**Relationship (*****n*** **=** **71)**		**Group differences**
	***n***	**%**	***n***	**%**	***n***	**%**	
Education^a^							χ^2^ = 8.85
Primary education	1	0.8	1	2.0	0	0.0	
VMBO	1	0.8	1	2.0	0	0.0	
HAVO	16	13.1	9	17.6	7	9.9	
VWO	21	17.2	7	13.7	14	19.7	
MBO	24	19.7	10	19.6	14	19.7	
HBO	25	20.5	11	21.6	14	19.7	
WO Bachelor	21	17.2	10	19.6	11	15.5	
WO Masters	13	10.7	2	3.9	11	15.5	

^a^* Education levels corresponding to the Dutch schooling system, presented in ascending order. VMBO, HAVO, and VWO represent higher secondary education, MBO represents intermediate vocational education, HBO represents university of applied science, and WO represents a university education*.

### Results

As described in the preregistration (https://osf.io/kfqw5), the original goal was to select 5–10 pictures of villains and 5–10 pictures of heroes. However, contrary to our expectations, we had to divert from the preregistered criteria because the TV characters were rather unknown. Ultimately, it was chosen to include the TV character if the combination of the following criteria were met: < 80% of the participants indicated not knowing the character, average physical appearance was rated > 5, preselected villains were averagely rated < 5 on the good/bad question, and preselected heroes were averagely rated > 5 on the good/bad question. This resulted in six villains and 10 heroes for the main study (see https://osf.io/2kcby/, for a codebook).

Moreover, we evaluated the mean physical appearance across all 28 heroic (*M* = 5.6, *SD* = 1.1) and 31 villainous TV characters (*M* = 4.7, *SD* = 1.2) using a paired samples *t*-test. We found that women averagely rated the heroes higher on romantic attraction than the villains, with *t*_(121)_ = −18.05, *p* < 0.001. This was not controlled for whether the participant knew the TV character, nor whether they thought he was good/bad. Besides, these differences occurred even for heroic and villainous characters played by the same actor (see Table 2). For example, Leonardo DiCaprio was included as villain Jordan Belfort (*The Wolf of Wall Street*), and as hero Jack Dawson (*Titanic*). As villain his physical appearance (*M* = 5.8, *SD* = 2.2) was evaluated significantly lower than as hero (*M* = 8.3, *SD* = 1.6), *t*_(121)_ = 13.80, *p* < 0.001.

**Table 2:** Physical appearance of TV characters (same actor) in Study 1 (Pilot Study).

**Actor**	**Villain**			**Hero**			**Mean Differences**
	**TV Character**	**Age**	**PA *M* (*SD*)**	**TV Character**	**Age**	**PA *M* (*SD*)**	
Zac Efron	Ted Bundy	27	6.2 (2.6)	Philip Carlyle	29	7.7 (1.6)	*t*_(121)_ = −7.38***
Leonardo DiCaprio	Jordan Belfort	30	5.8 (2.2)	Jack Dawson	20	8.3 (1.6)	*t*_(121)_ = −13.80***
Brad Pitt	Tyler Durden	30	5.9 (2.5)	Troy	30	6.5 (2.5)	*t*_(121)_ = −3.43**
Brad Pitt	Tyler Durden	30	5.9 (2.5)	Don Collier	30	5.2 (2.5)	*t*_(121)_ = 4.2***
Patrick Dempsey	Dominic Morgan	50	5.8 (2.3)	Derek Shephard	38	6.5 (2.2)	*t*_(121)_ = −4.15***
Chris Hemsworth	Billy Lee	35	4.5 (2.3)	Thor	25	6.8 (2.5)	*t*_(121)_ = −9.99***
Michael B. Jordan	Erik Killmonger	33	5.1 (2.3)	Adonis	35	7.2 (1.8)	*t*_(121)_ = −11.34***
Luke Evans	Gaston	35	4.9 (2.1)	John Moore	35	5.1 (2.3)	*t*_(121)_ = −0.83

*N = 122. Corresponding TV series/films are reported in the codebook (https://osf.io/bmtw5/). Age = (estimated) age of the TV character. PA = physical appearance on a scale from 0 (ugly) to 10 (handsome). It was not controlled for whether the women knew the TV character and rated him good/bad*.

**p < 0.01, ^***^p < 0.001.

### Discussion

The goal of Study 1 (pilot study) was to have a reliable picture selection of well-known villainous and heroic TV characters. We found that women generally rated the physical appearance of heroes higher than that of villains, regardless of whether the same actor played both a hero and villain role. This means that maybe picture characteristics (e.g., color, background) or physical aspects of the actor (e.g., clothing, haircut) have caused this discrepancy in physical appearance for the same actor. We also found that the recognizability of the TV characters was rather low. Participants did not have to answer the question of whether they thought the character was good/bad if they did not know him. Therefore, it is unknown whether the participants rated the villainous TV characters lower on physical appearance (than heroes) due to the character being a villain, or if there were other effects in play. For instance, although representing pictures of the TV characters were chosen, their backgrounds and poses differed. Besides, differences in age or ethnic background between the villainous and heroic TV characters played by the same actor might explain the differences in physical appearance.

Also, the findings regarding notoriety may interfere with Study 2, where we aim to measure romantic attraction to the personality of the TV character, which requires some knowledge about the TV character. Therefore, for Study 2 an extra option will be included to gain more insight in the notoriety of the TV characters. Even when participants do not know the TV characters, investigating romantic attraction (controlled for whether they think it is a hero/villain) can still provide useful information to unravel why some women are (not) attracted to dark personalities. This could be comparable to the initial romantic attraction in real life based on the first impression, which today can even be based on a picture when using online dating. Other studies have shown that when searching for potential partners online, the decision to “like” or “dislike” is primarily based on pictures [e.g., (48)]. While physical attractiveness is essential, other factors, such as perceived similarity, are just as important in selecting a potential partner online (48). Therefore, the use of pictures in the current study with a focus on the TV character's personality rather than his physical appearance could provide information about the underlying processes of romantic attraction.

## Study 2: Main Study

### Method

#### Participants and Procedure

Heterosexual women (*N* = 200) were approached online through convenience sampling to complete a Qualtrics questionnaire that allowed undergraduate students to obtain one course credit. Participants not studying at Tilburg University were not rewarded for participation. The questionnaire consisted of a picture-based experiment followed by self-report measurements, which took ~30–60 min in total. Participants were asked to sign an informed consent before participating, stating that they would rate TV characters on romantic attraction followed by questions about violence-related topics. It was emphasized that participation would be completely anonymous and voluntary, and that they could withdraw from participation at any time without providing explanation or consequences for course credit reimbursement. They were specifically asked whether they agreed that the data would be published openly (anonymously) after the data collection period. Inclusion criteria were women with heterosexual romantic preference, aged between 16 and 25 years, and Dutch-speaking.

Of these 200 women, four participants were removed due to > 25% missing data on all variables, and two participants were removed for not meeting the age criterion. This led to a final sample of 194 women, of whom 99 (51%) were currently in a romantic relationship, 94 were single (49%), and one (0.5%) did not reveal their relationship status. The mean age was 20.81 (*SD* = 2.42), ranging from 16 to 25 years. There were no significant differences between single women vs. in a relationship on education level (see Table 3, *p* = 0.265), but women in romantic relationships were significantly older than single women (see Table 4).

**Table 3:** Education level in Study 2 (Main Study).

	**Full sample** ***(N*** **=** **194)**		**Single** **(*****n*** **=** **94)**		**Relationship (*****n*** **=** **99)**		**Group differences**
	***n***	**%**	***n***	**%**	***n***	**%**	
Education^a^							χ^2^ = 16.85
VMBO	5	2.6	4	4.3	1	1.0	
HAVO	23	11.9	9	9.6	14	14.1	
VWO	79^b^	40.7	43	45.7	35	35.4	
MBO	21	10.8	14	14.9	7	7.1	
HBO	36	18.6	13	13.8	23	23.2	
WO Bachelor	14	6.2	6	6.4	8	8.2	
WO Masters	12	6.2	2	2.1	10	10.1	
Other	4	2.1	3	3.2	1	0.5	

^a^* Education levels corresponding to the Dutch schooling system, presented in ascending order. VMBO, HAVO, and VWO represent higher secondary education, MBO represents intermediate vocational education, HBO represents university of applied science, WO represents a university education, and Other (textual responses included: premaster, First Year's degree of HBO, etc.)*.

*^b^ One participant did not reveal her relationship status, but had a VWO education*.

**Table 4:** Descriptive statistics in Study 2 (Main Study).

	**Range**	**Full sample** ***(N*** **=** **194)**		**Single (*****n*** **=** **94)**		**Relationship (*****n*** **=** **99)**		**Group differences**
		***M***	***SD***	***M***	***SD***	***M***	***SD***	
Age (years)	16–25	20.81	2.42	20.41	2.40	21.21	2.83	*t*_(191)_ = −2.31*
Anxious	1–5	2.96	0.90	3.06	0.91	2.85	0.87	*t*_(191)_ = 1.61
Avoidant	1–4.5	2.55	0.75	2.77	0.79	2.34	0.66	*t*_(181.83)_ = 4.08***
YPI GM	0–2.1	0.48	0.42	0.54	0.46	0.42	0.36	*t*_(174.65)_ = 2.07*
YPI CU	0–2.2	0.50	0.42	0.58	0.48	0.43	0.34	*t*_(167.52)_ = 2.49*
YPI II	0.07–2.07	0.88	0.43	0.89	0.44	0.86	0.42	*t*_(191)_ = 0.48
ACV MF	1–2.7	1.11	0.31	1.14	0.33	1.09	0.29	*t*_(189)_ = 1.08
ACV FM	1–3	1.15	0.34	1.19	0.39	1.12	0.30	*t*_(168.44)_ = 1.24
ACV GD	1–2.8	1.20	0.36	1.23	0.40	1.17	0.32	*t*_(174.10)_ = 1.20
RA to Villains	0–9	3.94	1.81	4.16	1.97	3.72	1.60	*t*_(146)_ = 1.49
RA to Heroes	2.5–9.8	6.86	1.21	6.79	1.22	6.93	1.21	*t*_(159)_ = −0.77

*Anxious and Avoidant represent attachment styles indicated by the mean score on the corresponding dimensions of the RAAS (possible range: 1–5). YPI = Youth Psychopathic Traits Inventory (possible ranges: 0–3), factors: GM = Grandiose Manipulative; CU = Callous Unemotional; and II = Impulsive Irresponsible. ACV = Acceptance of Couple Violence (possible ranges: 1–4), subscales: MF = Male on Female Violence; FM = Female on Male Violence; GD = General Dating Violence. RA = Romantic Attraction (indicated as described in the methods section, with possible range: 0–10). One participant did not want to reveal their relationship status*.

^*^p < 0.05, ^***^p < 0.001.

#### Measures

##### Romantic Attraction

Prior to the self-report measurements, participants were shown 16 pictures from characters from TV series/movies. In total, they were shown six pictures of villains, and 10 pictures of heroes (see https://osf.io/d3hgb/, for a codebook), based on the selection of the pilot study. Because the notoriety of the TV characters was unexpectedly low, we included an additional option for the participants to indicate that they know the character a bit. Concretely, they were asked whether they know the character (1 = *yes*, 2 = *a bit*, or 3 = *no*), and to indicate whether they feel romantically attracted to the character on a scale from 0 (*repulsed*) to 10 (*attracted*). It was emphasized to indicate romantic attraction based on the TV character's personality rather than his physical appearance. After this part, participants were shown the pictures again and asked to rate the characters as good or bad on a scale from 0 (*villain*) to 10 (*hero*).

Before calculating the mean levels of romantic attraction to villains and heroes per participant, the notoriety of the TV characters was evaluated as indicated in the preregistration (https://osf.io/e8q6n). If > 40% of the participants did not know the character (answered with 3 = *no*), the TV character was excluded. However, because the required minimum was set at four (out of six) villains and seven (out of 10) heroes, the four best-known villains (see Table 5) and seven best-known heroes were chosen (see Table 6). Moreover, since the notoriety of the characters was too low to get the required number of participants, scenario G (all participants are included regardless of whether they know the character but controlled for their good/bad rating) was followed from the data exclusion scenarios described in the preregistration. Thus, for each participant and TV character, it was evaluated whether they rated the villain as a villain (< 5) and the hero as a hero (> 5) on their rating of each TV character as good or bad (0 = *villain*; 10 = *hero*), otherwise the response on romantic attraction for that character and participant was excluded. Then, the mean score for romantic attraction to villains and heroes (separately) was calculated if the participant had < 50 % missing data. This meant that a mean score for villains was calculated if the participant had scores on romantic attraction for ≥two out of four characters, and for heroes if the participant had scores on romantic attraction for ≥four out of seven characters (which was thus controlled for whether the participant correctly evaluated the villains as villain and heroes as hero). Mean scores on romantic attraction to villains could be calculated for 149 participants, and for heroes for 161 (of which both mean scores for romantic attraction to villains and heroes could be calculated for 131 participants).

**Table 5:** Final selection of included villainous TV characters in Study 2 (Main Study).

**TV Character**	**Age**	**Actor**	**Film/Series**	**% Unknown**	**RA *M* (*SD*)**	***n***
Joe Goldberg	30	Penn Badgley	You	22.7	3.1 (2.3)	140
Jordan Belfort	30	Leonardo DiCaprio	The Wolf of Wall Street	34.5	3.5 (2.3)	87
Don Massimo	29	Michele Morrone	365 Days	40.7	5.1 (2.8)	117
Tom Riddle	16	Christian Coulson	Harry Potter	42.8	3.2 (2.5)	123

*N = 194. Age = (estimated) age of the TV character. % Unknown = % of participants that answered “do you know this character?” with no (remaining participants answered with yes or a bit). RA = romantic attraction (scale: 0 = repulsed; 10 = attracted), means were calculated for the subsample (n) that rated the TV characters as villains (< 5)*.

**Table 6:** Final selection of included heroic TV characters in Study 2 (Main Study).

**TV Character**	**Age**	**Actor**	**Film/Series**	**% Unknown**	**RA *M* (*SD*)**	***n***
Will Turner	21	Orlando Bloom	Pirates of the Caribbean	36.1	6.6 (1.9)	135
Simon Basset	29	Regé-Jean Page	Bridgerton	39.2	7.1 (1.9)	127
Cedric Diggory	17	Robert Pattinson	Harry Potter	32.5	5.8 (2.4)	111
Derek Shepherd	38	Patrick Dempsey	Grey's Anatomy	40.7	6.8 (2.1)	159
Magic Mike	30	Channing Tatum	Magic Mike	21.6	6.7 (2.2)	129
Philip Carlyle	29	Zac Efron	The Greatest Showman	44.3	6.8 (1.9)	131
Jack Dawson	20	Leonardo DiCaprio	Titanic	8.8	7.8 (1.9)	178

*N = 194. Age = (estimated) age of the TV character. % Unknown = % of participants that answered “do you know this character?” with no (remaining participants answered with yes or a bit). RA = romantic attraction (scale: 0 = repulsed; 10 = attracted), means were calculated for the subsample (n) that rated the TV characters as heroes (>5)*.

The (estimated) mean age of the TV characters was comparable for the four included villains (*M* = 26.25, *SD* = 6.85) and seven included heroes (*M* = 26.29, *SD* = 7.30). The internal consistencies were calculated for each scale to provide indication of the reliability of the chosen pictures, which was found to be acceptable for both romantic attraction to villains (four pictures; α = 0.65; λ^2^ = 0.66) and romantic attraction to heroes (seven pictures; α = 0.61; λ^2^ = 0.64). However, given the large amount of missing data, these reliability estimations were based on 41 and 31 observations, respectively.

##### Revised Adult Attachment Scale (RAAS)

Adult attachment style was assessed using the Dutch translation of the RAAS (49, 50), which is an 18-item self-report measure to assess attachment related to close relationships. The scale encompasses three subscales: closeness (e.g., “I find it relatively easy to get close to people”), dependency (e.g., “I am comfortable depending on others”), and anxiety (e.g., “I often worry that other people don't really love me”), consisting of six items each. Participants were asked how they generally feel about each statement concerning close relationships, rated on a 5-point Likert scale (1 = *not at all characteristic of me*, to 5 = *very characteristic of me*). The scores on these 18 items can also be used to compute two attachment dimensions, avoidance (closeness and dependency) and anxiety (anxiety). Low scores on avoidance and anxiety dimensions represent an indication of secure attachment style, while higher scores represent a more insecure attachment style. The RAAS subscales are considered sufficiently reliable [closeness: α = 0.77; dependency: α = 0.80; anxiety: α = 0.86 (51)]. In the current study, the internal consistency of both the avoidance (α = 0.89; λ^2^ = 0.90) and anxiety dimension (α = 0.85; λ^2^ = 0.86) were considered good.

##### Youth Psychopathic Traits Inventory (YPI)

The YPI (52) is a 50-item self-report measure to assess psychopathic personality traits in the general population, of which the Dutch translation was used (53). A common problem when measuring psychopathic traits is social desirability, which is lowered in the YPI by presenting the traits positively/praiseworthy (52). Participants were asked how each statement generally applied to them (e.g., “I am better than everyone on almost everything”), rated on a 4-point Likert scale (0 = *does not apply*, to 3 = *applies very well*). The YPI encompasses 10 subscales (Dishonest Charm, Grandiosity, Lying, Manipulation, Callousness, Unemotionality, Remorselessness, Impulsivity, Thrill-Seeking, and Irresponsibility), forming three factors: Grandiose-Manipulative (GM), Callous-Unemotional (CU), and Impulsive-Irresponsible (II). However, it is suggested to primarily focus on the YPI total score in the general population (54). A high total score indicates a high level of psychopathic personality traits. The YPI total score shows acceptable reliability in female adolescents (*M*_*age*_ = 15.6) assessed in the general population [α = 0.74 (55)]. Besides, the YPI items are considered to be measurement invariant for boys and girls (54). In this study, the internal consistency was excellent for the YPI total score (α = 0.92; λ^2^ = 0.93). Since the three factors are used to estimate the latent variable indicating maladaptive personality traits, the internal consistencies were additionally calculated for the three factors, which were considered excellent for GM (α = 0.90; λ^2^ = 0.91), and good for CU (α = 0.84; λ^2^ = 0.85) and II (α = 0.81; λ^2^ = 0.82).

##### Acceptance of Couple Violence (ACV)

The ACV is an 11-item self-report measure to assess acceptance of dating violence (56). It consists of three subscales: Acceptance of Male on Female Violence (MF; e.g., “A boy angry enough to hit his girlfriend must love her very much”), Female on Male Violence (FM; e.g., “Boys sometimes deserve to be hit by the girls they date”), and General Dating Violence (GD; e.g., “Violence between dating partners can improve the relationship”). For this study, items were translated into Dutch by two independent researchers, and back translated into English by a third. Participants were asked to indicate to what extent the statements correspond to their beliefs, rated on a 4-point Likert scale (1 = *strongly disagree*, 2 = *disagree*, 3 = *agree*, 4 = *strongly agree*), where higher scores implied greater acceptance of violence. The reliability is considered good for the subscales MF (α = 0.71), FM (α = 0.77), and GD (α = 0.81) measured in adolescents [mean age = 15 (57)]. In the current study, the internal consistency for the total ACV was considered good (α = 0.88; λ^2^ = 0.88) and the same accounted for the subscales (MF: α = 0.75, λ^2^ = 0.76; FM: α = 0.71, λ^2^ = 0.72; and GD: α = 0.75; λ^2^ = 0.77).

#### Statistical Analyses

The descriptive and correlational analyses were performed in SPSS version 26.0. Mean scores on romantic attraction were calculated if the participant had < 50% missing values for villains and heroes separately (final number of missing values on the mean romantic attraction score: villains = 45, and heroes = 33). Then, after inspection of the missing values on all self-report items (YPI, RAAS, and ACV) and picture-related romantic attraction rating (after controlling for the hero/villain evaluation) on item/picture-level, using Little's missing completely at random (MCAR) test, it was found that the missing values did not occur randomly, χ^2^ (12,480, *N* = 194) = 12,781.18, *p* = 0.029 (58). Given the expected high number of missing values related to the notoriety of the TV characters and its consequences for the assessment of romantic attraction (controlled for villain/hero evaluation), imputation was not chosen. There are several reasons that could logically have influenced these missing values, which we will elaborate on in the discussion. Outliers were examined using the interquartile range (IQR) to identify extreme values, scores >1.5 ^*^ IQR below the first quartile or beyond the third quartile were considered outliers (59). In total, 75 participants had outliers on one (or more) of the measured variables, but as described in the preregistration outliers were expected and ignored. Independent *t*-tests were conducted to evaluate whether there were statistically significant differences between single women and women in a relationship. In addition, correlations between the variables of interest were evaluated for the entire sample using the Pearson correlation coefficient (*p* < 0.05).

The analyses to evaluate the models were computed in R using package “lavaan” (60). SEM analysis was applied to examine the effects of attachment, maladaptive personality traits, and acceptance of couple violence, on romantic attraction to villains and heroes. To obtain sufficient power based on the hypothesized theoretical model we aimed to acquire *N* ≥ 300 given the generally accepted rule of thumb: 10 observations per estimated parameter (61). The minimally required sample size was set to *N* ≥ 150, based on the ratio: five observations per estimated parameter (62); and the proposed minimum (*N* ≥ 150) for performing a simple confirmatory factor analysis (CFA) model without missing values (63). However, the current sample resulted in 130 participants that could be included in the SEM model, excluding participants for whom missing values occurred on the included variables.

The variables avoidant and anxious attachment were entered into the model as observed values. Maladaptive personality traits and acceptance of couple violence were entered as latent variables, each with three underlying factors (respectively: GM, CU, and II; and MF, FM, and GD). Romantic attraction to villains and heroes were included as observed values. To estimate the model, Maximum Likelihood (ML) estimation was used. Although ML is fairly robust against violations of normality, the normal distribution of the outcome variables was assessed using the following rules of thumb: skewness ≤ 2, and kurtosis ≤ 7 (64), which did not indicate non-normality (romantic attraction to villains: skewness = 0.35 and kurtosis = 2.84; to heroes: skewness = −0.76 and kurtosis = 4.10). Model fit was evaluated using χ^2^ (*p* ≥ 0.05), Root Mean Square Error of Approximation (RMSEA; value < 0.08), Comparative Fit Index (CFI; value > 0.90), and Standardized Root Mean Square Residual (SRMR; value < 0.08) (65).

### Results

#### Descriptive and Correlational Analyses

Following the descriptive analyses as depicted in Table 4, single women scored significantly higher on avoidant attachment style and the YPI, GM, and CU factors than women in a relationship. When comparing the mean scores on romantic attraction to villains and to heroes (for *n* = 131 women with mean scores on both villain/hero scales), women are generally significantly more romantically attracted to heroes (*M* = 6.9, *SD* = 1.2) than to villains (*M* = 4.1, = 1.7), with *t*_(130)_ = −17.39, *p* < 0.001. For single women and women in a relationship only, we found the same differences. Romantic attraction to heroes was higher for both single women (*n* = 71, *M* = 6.8, *SD* = 1.2) and women in a relationship (*n* = 60, *M* = 6.9, *SD* = 1.2) compared to romantic attraction to villains (single women: *M* = 4.3, *SD* = 1.9; women in a relationship: *M* = 3.8, *SD* = 1.4), with for single women *t*_(70)_ = −10.67, *p* < 0.001; and for women in a relationship *t*_(59)_ = −15.22, *p* < 0.001. Moreover, as presented in Table 7, for the entire sample only the YPI GM factor was found to be statistically significantly (positively) correlated with outcome variable romantic attraction to villains (*r* = 0.20, *n* = 149, *p* = 0.013). For outcome variable romantic attraction to heroes, the only statistically significant negative correlations were found for avoidant attachment style (*r* = −0.16, *n* = 161*, p* = 0.038), the YPI CU factor (*r* = −0.19, *n* = 161, *p* = 0.016), and positive for the YPI II factor (*r* = 0.21, *n* = 161, *p* = 0.007). Regarding the intercorrelations of the predictors, statistically significant positive correlations were found between anxious attachment style and acceptance of general dating violence, between avoidant attachment style and all three YPI factors, and between all three YPI factors and all three ACV subscales (see Table 7).

**Table 7:** Intercorrelations for study variables in Study 2 (Main Study).

**Variable**	**1**	**2**	**3**	**4**	**5**	**6**	**7**	**8**	**9**	**10**
1. Romantic Attraction to Villains	–									
2. Romantic Attraction to Heroes	−0.23**	–								
3. Anxious Attachment	0.02	0.02	–							
4. Avoidant Attachment	0.01	−0.16*	0.62***	–						
5. YPI Grandiose Manipulative	0.20*	0.06	0.10	0.29***	–					
6. YPI Callous Unemotional	0.08	−0.19*	0.08	0.40***	0.60***	–				
7. YPI Impulsive Irresponsible	−0.01	0.21**	0.14	0.15*	0.53***	0.40***	–			
8. ACV Male on Female	−0.03	−0.01	0.03	0.08	0.30***	0.33***	0.17*	–		
9. ACV Female on Male	0.003	0.01	0.10	0.08	0.28***	0.31***	0.19**	0.80***	–	
10. ACV General Dating	−0.003	0.002	0.14*	0.14	0.28**	0.31***	0.22**	0.67***	0.66***	–

*N = 194. Due to missing values, adjusted sample sizes were used for some of the variables (minimum: n = 131)*.

^*^p < 0.05, ^**^p < 0.01, ^***^p < 0.001.

#### SEM Analyses

CFA was used to estimate the conceptual models. The latent variables were identified by fixing the variance of each latent variable to one, leaving all loadings of the indicators free. For a better fitting model, *post-hoc* modification was applied to make changes to the original hypothesized model. The correlations between the YPI factors and ACV subscales were quite strong, which makes sense within the theoretical intergenerational transmission of violence framework (16). Besides, maladaptive personality traits—specifically narcissism—have been related to more acceptance of violence in general (66). Thus, based on the modification indice of 12.08, covariance was added between the latent variables (maladaptive personality traits and acceptance of couple violence). The model was assessed for model fit, which resulted in a significant χ^2^(24) = 48.811, *p* = 0.002, and RMSEA of 0.089 (CI 90% [0.053; 0.125]) indicating that the model does not fit the data exactly. However, χ^2^ (which is also used in RMSEA) can be easily biased by a low sample size resulting in reduced power to detect model fit (as was the case with *n* = 130) (65, 67, 68). Therefore, based on the CFI (0.933) and SRMR (0.055) implying good absolute and incremental fit, the proposed model was accepted. The included predictors together explained 6.9% of the variance in romantic attraction to villains and 10.1% of the variance in romantic attraction to heroes.

**Figure 2:**
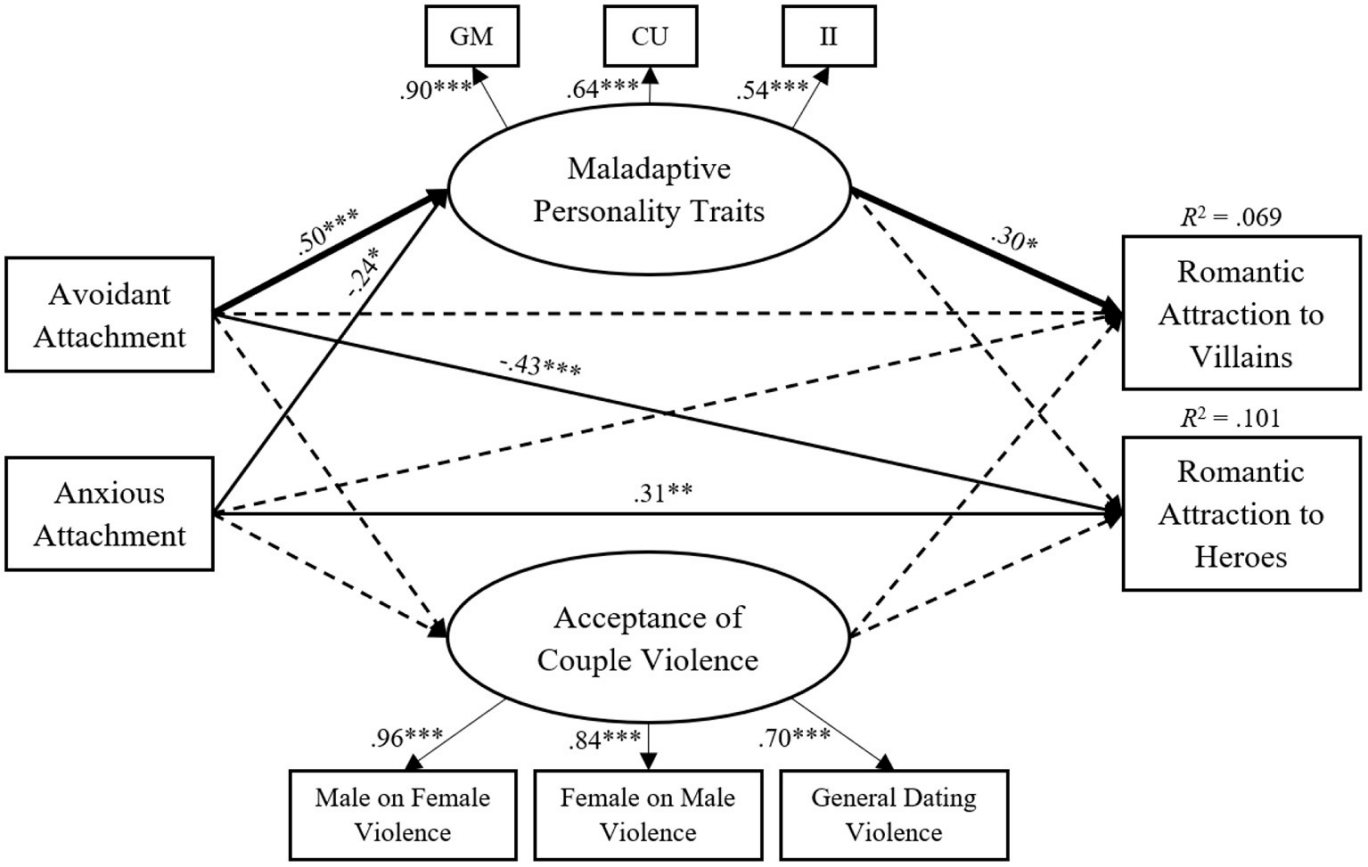
Estimated model for predicting romantic attraction to villains. Standardized estimates of path coefficients. Dashed lines represent nonsignificant relations, and the bold line represents a significant indirect path. *p < 0.05, **p < 0.01, ***p < 0.001.

As presented in Figure 2 and Table 8, avoidant attachment style significantly negatively predicted romantic attraction to heroes (path c2: β = −0.43, *p* < 0.001), while anxious attachment style significantly positively predicted romantic attraction to heroes (path c4: β = 0.31, *p* = 0.005). Besides, avoidant attachment style had a significant positive direct effect on maladaptive personality traits (path a1: β = 0.50, *p* < 0.001), and anxious attachment style had a significant negative direct effect on maladaptive personality traits (path a2: β = −0.24, *p* = 0.038). Consequently, maladaptive personality traits significantly negatively predicted romantic attraction to villains (path b1: β = 0.30, *p* = 0.010). Furthermore, there was a significant positive indirect effect of avoidant attachment style on romantic attraction to villains through maladaptive personality traits (indirect effect a1^*^b1: β = 0.15, *p* = 0.029), indicating a full mediation. No other significant indirect effects were found.

**Table 8:** Unstandardized and standardized model results in Study 2 (Main Study).

	**Estimate**	***SE***	**Std. all**	***p***
**Romantic Attraction to Villains**				
Avoidant Attachment (path c1)	−0.161	0.281	−0.071	0.568
Anxious Attachment (path c3)	0.081	0.213	0.043	0.705
Maladaptive Personality Traits (path b1)	0.460	0.178	0.295	**0.010**
Acceptance of Couple Violence (path b3)	−0.202	0.165	−0.120	0.220
**Romantic Attraction to Heroes**				
Avoidant Attachment (path c2)	−0.682	0.194	−0.425	**<** **0.001**
Anxious Attachment (path c4)	0.410	0.147	0.308	**0.005**
Maladaptive Personality Traits (path b2)	0.239	0.122	0.216	0.050
Acceptance of Couple Violence (path b4)	−0.120	0.114	−0.100	0.294
**Maladaptive Personality Traits**				
Avoidant Attachment (path a1)	0.721	0.179	0.496	**<** **0.001**
Anxious Attachment (path a2)	−0.293	0.141	−0.243	**0.038**
**Acceptance of Couple Violence**				
Avoidant Attachment (path a3)	0.024	0.157	0.018	0.880
Anxious Attachment (path a4)	−0.114	0.130	−0.102	0.381
**Covariances**				
Maladaptive Personality Traits and Acceptance of Couple Violence	0.361	0.091	0.361	**<** **0.001**
Romantic Attraction to Villains and Romantic Attraction to Heroes	0.425	0.170	0.229	**0.013**
Indirect effect 1 (path a1*b1)	0.332	0.151	0.146	**0.029**
Indirect effect 2 (path a1*b2)	0.172	0.097	0.107	0.077
Indirect effect 3 (path a2*b1)	−0.135	0.083	−0.072	0.104
Indirect effect 4 (path a2*b2)	−0.070	0.049	−0.053	0.153
Indirect effect 5 (path a3*b3)	−0.005	0.032	−0.002	0.881
Indirect effect 6 (path a3*b4)	−0.003	0.019	−0.002	0.882
Indirect effect 7 (path a4*b3)	0.023	0.032	0.012	0.475
Indirect effect 8 (path a4*b4)	0.014	0.020	0.010	0.501

*N = 130. SE = standard error; Std. all = all variables are standardized. Significant p-values (< 0.05) are in bold*.

To assess whether the direct paths to romantic attraction to villains and heroes were significantly different, an additional constrained model was tested where these paths were constrained (b1 = b2, b3 = b4, c1 = c2, and c3 = c4). These constraints did not lead to significantly increased model fit χ^2^(4) = 7.64, *p* = 0.106. Therefore, these paths and predictors of romantic attraction to villains and heroes were interpreted to be different from each other.

### Discussion

The goal of Study 2 (main study) was to investigate potential differences in predictors for romantic attraction to villains and heroes. We found that women are more romantically attracted to heroes than villains, which is in line with previously mentioned findings from prior research (30, 31, 35, 40, 43), but also with the pilot study. These findings occurred even despite our efforts to reduce potentially perceived self-threat by using pictures of fictional characters (44), and attempts to include approximately equally handsome TV characters based on the pilot study. Overall, the proposed theoretical model fits with the estimated SEM model, however, not all hypothesized paths were supported. For villains, it was found that maladaptive personality traits positively predicted romantic attraction to villains. Avoidant attachment was also found to be positively related to romantic attraction to villains, but only through the full mediation of maladaptive personality traits. For heroes, merely direct—yet opposite—effects of attachment style on romantic attraction to heroes were found. More specifically, avoidant attachment style negatively predicted romantic attraction to heroes, and anxious attachment style positively predicted romantic attraction to heroes. This could lead to the conclusion that there are indeed different predictors for romantic attraction to villains and heroes.

The positive association between maladaptive personality traits and romantic attraction to villains can be explained by the similarity principle (40, 41), which was also found by Sleep et al. (30). On the contrary, we did not find an (opposite, hence negative) effect of maladaptive traits on romantic attraction to heroes. However, a potential explanation could be that these heroic (good) TV characters would not specifically score lower on maladaptive personality traits, as some psychopathic traits also positively correlate with heroic acts (39). Moreover, the finding that attachment style is related to romantic attraction, and specifically at this early stage (initial romantic attraction by simply seeing another person), is in concordance with the theoretical framework of the attachment theory (20, 24, 69). Though, we did not expect these different effects for the separate attachment styles, because for both, higher scores are indicative of a more insecure attachment style (24, 49). More specifically, our findings directly contradict the previously found positive relation between psychopathic traits and anxious attachment style in women, which was explained by evolutionary motives (70). Therefore, the relation between attachment style, maladaptive personality traits, and romantic attraction might be more complicated.

We found no relations between acceptance of couple violence and the other variables in the estimated model, but we did find correlations with maladaptive personality traits. In the current sample, the means on the ACV subscales were rather low which implies that participants were rather unaccepting of couple violence. This can be explained by the relatively young sample of women, who might not have had many romantic relationships. It could also be a consequence of social desirability bias or actual low acceptance rates, given the increased societal awareness of IPV that prevent violence (71). In the Netherlands, there are several national government-funded campaigns to raise awareness for domestic violence targeted at among others victims, bystanders, and children (e.g., https://veiligthuis.nl). Therefore, these Dutch-speaking women might be relatively less accepting of couple violence or may have responded more socially desirable.

Furthermore, we found mean differences for women depending on their relationship status. Single women had a relatively more avoidant attachment style than women in a relationship, scored higher on GM and CU traits, and were somewhat younger. Although these constructs are relatively stable (21), it could be argued that maladaptive personality traits and avoidant attachment style slightly decrease when someone is in a romantic relationship, due to achieved safety or a sense of connectedness (40). On the contrary, one could also say that the higher levels of maladaptive personality traits and avoidant attachment style are the reason that they are not in a romantic relationship. This can be explained by previous findings that maladaptive personality traits are negatively related to relationship satisfaction (72), however, this does not inform about the direction of effects.

## General Discussion

The goal of the current studies was to unravel the effects of adult attachment styles, maladaptive personality traits, and couple violence acceptance in predicting romantic attraction to villains and heroes, to gain more insight into whether and why women are romantically attracted to bad or good guys. We found different predictors and effects for romantic attraction to heroes and villains. Adult attachment styles contribute (in)directly to the prediction of romantic attraction and maladaptive personality traits, but not to acceptance of couple violence. Most of the findings can be explained with the similarity principle (40, 41), meaning that for example more maladaptive personality traits are related to more romantic attraction to villains (for whom we also assume more maladaptive personality traits).

However, different complex mechanisms seem to apply to attachment styles. We found a fairly strong (positive) correlation between anxious and avoidant attachment styles, on which participants can score high simultaneously. Though, the estimated model revealed different (opposite) effects of the attachment styles on romantic attraction. This suggests that these findings might be more complicated and the attachment styles might even interact, as was previously found for predicting attraction to humor styles (73). In the current study, anxious attachment style represented the internal working model of the self, while avoidant attachment style represented the internal working model of the other (24). An anxious attachment style can also incorporate fear of abandonment (25) and is related to low self-esteem (74). The mechanism behind this is suggested to be hyperactivation of the attachment system, meaning that anxiously attached adults are actively looking for connectedness with others (24, 75). In this light, the positive relation between anxious attachment style and romantic attraction to heroes can be explained by the idea that more anxiously attached women might be more desperately in need of a romantic relationship than women with lower levels of anxious attachment. This could in turn lead to a higher romantic attraction to the heroic TV characters. On the contrary, women with higher scores on avoidant attachment style are more prone to refrain from seeking help (25) and lack a need for emotional intimacy (76) through deactivation of the attachment system (75). This means that avoidantly attached adults strive for independence from others (24), and therefore might be less eager to engage in a romantic relationship than women with lower levels of avoidant attachment. This could explain the negative relation between avoidant attachment style and romantic attraction to heroes. Though, the opposite—positive—relation between avoidant attachment style and romantic attraction to villains (fully mediated by maladaptive personality traits) indicates that this romantic attraction might be dependent of the potential partner. Perhaps more avoidantly attached women are indeed more romantically attracted to villains than heroes, which consequently might be a risk factor for IPV victimization.

As mentioned earlier, both anxious and avoidant attachment styles have been related to IPV victimization (25), though mainly in describing why women stay in abusive relationships. Anxiously attached females might have a more negative self-image and view themselves as unworthy of love, which has been suggested to function as a continuation of the violent dynamics within a relationship with IPV. For more avoidantly attached females, it is suggested that the continuation of abuse might be caused by a lack of social support or connectedness that disables them to seek help (25). From another related perspective, the association between IPV victimization and insecure attachment styles might be trough communication and conflict styles, especially for an anxious attachment style (77). The dynamics within an abusive relationship are probably more complicated since violent partners are often very charming at the beginning of the relationship, but increasingly isolate the victim from their social network (78, 79). In extreme cases, the abusive partner might even use coercive and controlling behaviors (e.g., manipulation) to make sure the victim will stay, which can be seen as intimate terrorism (80). Therefore, more research is needed to explore these associations between attachment and IPV victimization, and particularly the underlying processes.

Furthermore, although acceptance of couple violence was unrelated to romantic attraction, this does not mean that acceptance of couple violence is unrelated to IPV involvement, given the correlations found with maladaptive personality traits. Maybe women at increased risk of becoming a victim of IPV are not particularly attracted to dark personalities or villains, but there are social risk factors that increase the likelihood of IPV involvement (16). The current sample included relatively highly-educated women from a developed country (i.e., the Netherlands), while in other countries and cultures, violent behavior, such as disciplining a woman, is a man's right and culturally accepted (71). Besides, women involved in IPV might not even accept couple violence, but there may be other processes involved that explain why they become a victim. Following Aker's social learning theory, the likelihood of criminal behavior is determined by the interplay of four theoretical concepts: differential association (i.e., exposure to behavioral and normative definitions of violence), definitions (i.e., moral attitudes/orientations toward violence), imitation (i.e., replicating observed behavior), and differential reinforcement (i.e., operant conditioning) (81). This was applied to the context of IPV perpetration, where it was found that the constructs definitions and imitation were not related to IPV perpetration, while differential association and reinforcement were (82). This suggests that attitude toward violence (definitions) might not play as big a role as expected for IPV perpetration, which might also account for IPV victimization. Likewise, there might be a coercive dominant socialization process leading young women to connect violence with attraction (38). Thus, the interpersonal processes involved might encompass more than just attitudes toward violence, potentially explaining why we found no associations between acceptance of couple violence and romantic attraction.

### Strengths, Limitations, and Future Directions

One of the major strengths of the current study design is the use of a novel approach to investigate romantic attraction by using pictures of TV characters rather than a speed-date paradigm or personality vignettes. A pilot study was conducted to increase the reliability of the picture selection, both studies were preregistered, and the data was openly published. However, there were also some limitations that should be considered. First, the notoriety of the TV characters was lower than originally expected. As a result, both studies had to divert from the preregistered criteria. In addition, the variety between the TV characters and their pictures was rather high. Although the internal consistencies of the scales of pictures (villains/heroes) were acceptable, these could only be calculated from rather small subsamples of participants who had no missing values. We calculated a mean score if the participants scored at least 50% of the characters, meaning that different participants could have rated different characters and still got a mean score. Therefore, these scores on romantic attraction between participants may not be as comparable as intended. Besides, some characters might have been rated more attractive because of the picture that was shown, as some characters looked directly into the camera while others looked away, backgrounds differed, etc. Despite the preselection of the pilot study (selected TV characters in the main study were rated > 5 on physical appearance) and the focus on the character's personality over his appearance, we cannot be sure that physical appearance did not play a role. There may be alternative explanations based on physical characteristics (e.g., hair color, eyes, etc.). Besides, although a TV character might be overall good/bad, this does not mean that the TV character solely shows good/bad behavior throughout the series/film. Therefore, the participant could be thinking of other aspects or scenes for which the TV character might be known. Although this was controlled for by asking the participant whether they thought the TV character was good or bad, we cannot know what specific characteristic(s) the romantic attraction was based on.

The sample size used for the SEM analyses was rather low and therefore we did not have optimal statistical power. The missing values of the participants were quite extensive and did not occur randomly, which could be explained by preference of films/series depending on attachment style, maladaptive personality traits, or acceptance of couple violence. Another explanation could be that some participants rated the TV character less bad because they were romantically attracted to him or thought he was handsome. This could have resulted in exclusion of that score while it pinpoints an important potential mechanism that can explain why women are more romantically attracted to a villain.

For future studies, we recommend including measures of childhood trauma and IPV. It would be very informative to include victimized women of IPV and compare them to the general population, as these effects might differ and there might be more variation in the acceptance of couple violence. The current study design assumed developmental pathways between the constructs but was cross-sectional and therefore failed to measure these longitudinal effects, which is recommended for future research. More specifically, relationship history of the participants could play an important role, it would therefore be informative to include the development of longitudinal relationships and satisfaction related to IPV. Another potentially informative study design would be to include pictures of actual (non-)violent offenders and men from the general population, to compare whether women are also less romantically attracted to real-life offenders rather than to fictional villains. However, we cannot divert from physical characteristics whether someone is a criminal, like Lombroso theorized (83). Finally, the current study focused exclusively on women, while men can also be victims of IPV, which has been largely understudied (3).

## Conclusion

Despite the complications of this innovative picture-based research design to investigate romantic attraction, different predictors were found for romantic attraction to villains and heroes. This indicates that there are individual differences in the preference for a potential romantic partner that may be related to attachment style and/or maladaptive personality traits. This may explain why some women are relatively more romantically attracted to villains, although the majority of women in the study is romantically attracted to heroes. However, our results require caution to generalize the findings to daily life. Therefore, future studies are needed to replicate this study and further disentangle underlying processes of romantic attraction.

## Author's Note

Jaime Lannister tells his niece/daughter Myrcella Baratheon “We don't choose whom we love. It just well, it's beyond our control.”, Game of Thrones (84).

## Data Availability Statement

The datasets presented in this study can be found in online repositories. The names of the repository/repositories and accession number(s) can be found at: https://osf.io/bmtw5/ (Project 2: Romantic Attraction to Villains).

## Ethics Statement

The studies involving human participants were reviewed and approved by Ethics Review Board of Tilburg School of Social and Behavioral Sciences. The participants provided their written informed consent to participate in this study.

## Author Contributions

IF and LV conceived the presented idea and collected the data under supervision of SB. IF wrote the manuscript. All authors provided critical feedback and helped shape the research, analysis, and manuscript.

## Conflict of Interest

The authors declare that the research was conducted in the absence of any commercial or financial relationships that could be construed as a potential conflict of interest.

## Publisher's Note

All claims expressed in this article are solely those of the authors and do not necessarily represent those of their affiliated organizations, or those of the publisher, the editors and the reviewers. Any product that may be evaluated in this article, or claim that may be made by its manufacturer, is not guaranteed or endorsed by the publisher.
